# Synthesis, Characterization and Enhanced Sensing Properties of a NiO/ZnO p–n Junctions Sensor for the SF_6_ Decomposition Byproducts SO_2_, SO_2_F_2_, and SOF_2_

**DOI:** 10.3390/s17040913

**Published:** 2017-04-21

**Authors:** Hongcheng Liu, Qu Zhou, Qingyan Zhang, Changxiang Hong, Lingna Xu, Lingfeng Jin, Weigen Chen

**Affiliations:** 1 College of Engineering and Technology, Southwest University, Chongqing 400715, China; swulhcx@163.com (H.L.); zqyswu@163.com (Q.Z.); hcx111000@163.com (C.H.); lingnaxu@cqu.edu.cn (L.X.); 2 State Key Laboratory of Power Transmission Equipment & System Security and New Technology, Chongqing University, Chongqing 400030, China; cqujlf@cqu.edu.cn (L.J.); weigench@cqu.edu.cn (W.C.)

**Keywords:** synthesis and characterization, NiO-decorated ZnO, p–n junctions, sensing properties, SF_6_ decomposition byproducts

## Abstract

The detection of partial discharge and analysis of the composition and content of sulfur hexafluoride SF_6_ gas components are important to evaluate the operating state and insulation level of gas-insulated switchgear (GIS) equipment. This paper reported a novel sensing material made of pure ZnO and NiO-decorated ZnO nanoflowers which were synthesized by a facile and environment friendly hydrothermal process for the detection of SF_6_ decomposition byproducts. X-ray diffraction (XRD), field emission scanning electron microscopy (FESEM), transmission electron microscopy (TEM), high resolution transmission electron microscopy (HRTEM), energy-dispersive X-ray spectroscopy (EDS) and X-ray photoelectron spectroscopy (XPS) were used to characterize the structural and morphological properties of the prepared gas-sensitive materials. Planar-type chemical gas sensors were fabricated and their gas sensing performances toward the SF_6_ decomposition byproducts SO_2_, SO_2_F_2_, and SOF_2_ were systemically investigated. Interestingly, the sensing behaviors of the fabricated ZnO nanoflowers-based sensor to SO_2_, SO_2_F_2_, and SOF_2_ gases can be obviously enhanced in terms of lower optimal operating temperature, higher gas response and shorter response-recovery time by introducing NiO. Finally, a possible gas sensing mechanism for the formation of the p–n junctions between NiO and ZnO is proposed to explain the enhanced gas response. All results demonstrate a promising approach to fabricate high-performance gas sensors to detect SF_6_ decomposition byproducts.

## 1. Introduction

Gas-insulated switchgear (GIS) is used extensively In electrical power systems, where it presents unique advantages, including small space occupation, low failure rates, outstanding breaking capacity, and so on [1,2]. In the event of a short circuit in big systems, the high-voltage circuit breaker (CB) in the switchgear is the last line of defense, so the GIS must remain healthy [3]. Sulfur hexafluoride (SF_6_) as a widely used insulating medium in GIS due to its excellent insulating and arc-extinguishing properties [4,5]. Despite of the high reliability of GIS equipment, some internal defects could still lead to different degrees of partial discharge (PD) when the GIS equipment has been running for a long time [6]. When exposed to the energy of partial discharges, the SF_6_ gas in GIS equipment could decompose and generate SF_4_, SF_3_, and SF_2_ [7], as well as various low-fluorine sulfides. The decomposed SF_6_ reacts further with trace moisture, oxygen and solid materials, thus producing multiple chemical products (SOF_4_, SOF_2_, SO_2_F_2_, SO_2_, H_2_S and HF, among others) [8,9]. These products accelerate the aging of the insulation and the corrosion of metal surfaces, which may eventually lead to GIS insulation faults. Some new studies show that the composition and content of these byproducts could be used to evaluate the operating state and insulation level of GIS equipment [10,11]. Consequently, many methods such as infrared absorption spectrometry [12], photoacoustic (PA) spectroscopy technology [13], and test tube method [14] have been used to detect and analyze the partial discharge decomposition products of SF_6_. However, these methods are offline laboratory detection methods and it is difficult to achieve on-line monitoring.

Recently, metal-oxide semiconductor materials with low operating temperature, good gas responses and stable properties have attracted much research interest for various applications, including gas sensors [15,16], solar cells [17], UV sensors [18]. Particularly zinc oxide (ZnO) [19,20,21,22], a typical n-type semiconducting material with a wide band gap, that can easily form p–n junctions with NiO has attracted the attention of researchers [23,24,25]. Liu et al. reported p-NiO/n-ZnO hetero-structures synthesized by a low-cost and environmentally friendly strategy and investigated their gas sensing performance to acetone [26]. Using a simple photochemical deposition method Dai et al. prepared honeycomb-like NiO/ZnO hetero-structured nanorods exhibiting dramatically enhanced UV detection performance [27]. Tian et al. developed a hydrothermal method followed by calcination to prepare NiO/ZnO p–n heterostructures which exhibited high responses to ethanol vapor [28]. However, to the best of our knowledge, the hydrothermal synthesis of p-NiO/n-ZnO composite nanostructures for SF_6_ decomposed components gas sensors has rarely been reported.

Thus, in this context we have successfully synthesized pure ZnO and NiO-decorated ZnO flower-like nanometer materials and systematically researched their gas sensing performances toward the SF_6_ decomposition byproducts SOF_2_, SO_2_F_2_, and SO_2_. By a comparative study, we conclude that the introduction of NiO plays an important role in improving the sensing performances of pure ZnO microflowers toward the main components of SF_6_ decomposition, in terms of lower optimal working temperature, higher gas response and shorter response-recovery time. Moreover, a possible sensing mechanism to explain this interesting performance is also proposed, that is, the formation of p–n junctions NiO and ZnO. These results demonstrate a promising approach to fabricate gas sensors to detect SF_6_ decomposed components in GIS.

## 2. Experimental Details

### 2.1. Preparation and Characterization of Materials

All the chemical reagents were analytical-grade, purchased from Beijing Chemicals Co. Ltd. (Beijing, China) and used as received without any further purification. Pure ZnO flower-like nanometer materials were synthesized by a facile and environmentally friendly hydrothermal method. In a typical procedure, Zn(NO_3_)_2_·6H_2_O (10 mmol) was firstly dissolved completely in a mixed solution distilled water and anhydrous ethanol (40 mL of each) to form a precursor solution. After magnetic stirring for about 30 min, the mixture was transferred into a 100 mL Teflon-lined stainless steel autoclave, and maintained at 180 °C for 12 h in order to obtain flower-like nanomaterials. Finally, the collected products were washed with distilled water and anhydrous ethanol several times, and dried at 80 °C for 8 h to further use. 3 at-% NiO-decorated ZnO flower-like sensing materials were synthesized by the same process mentioned above, except a certain proportion of Ni(NO_3_)_2_·6H_2_O was added to the precursor solution.

The crystalline parameters of the synthesized sensing materials were characterized by X-ray diffraction (XRD) with Cu Kα radiation (40 kV, 200 mA and λ = 1.5418 Å). Field emission scanning electron microscopy (FESEM, FEI, Hillsboro, OR, USA; operated at 10 kV), transmission electron microscopy (G2 F20 S-TWIN, Tecnai, Hillsboro, OR, USA; operated at 200 kV) and HRTEM combined with select area electron diffraction (SAED) were used to examine the morphology of the products. The chemical compositions of the prepared samples were conducted using energy-dispersive X-ray spectroscopy (EDS). X-ray photoelectron spectroscopy (XPS) was performed on an ESCLAB MKII using monochromatic Al Kα as the X-ray exciting source to investigate the chemical state of elements existing in the products.

### 2.2. The Fabrication of Sensors

**Figure 1:**
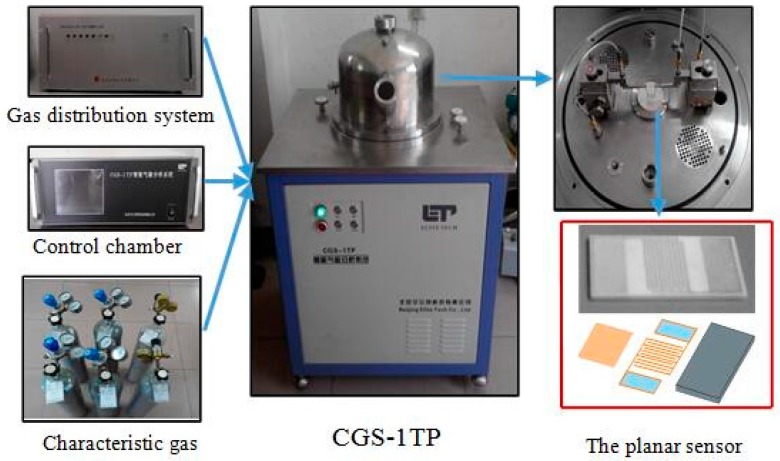
Schematic diagram of the CGS-1TP gas sensing analysis system.

Gas sensors were fabricated by a screen-printing technique with planar ceramic substrates [29] Figure 1 (the planar sensor) shows a structural drawing of the sensor, which mainly consists of three parts: ceramic substrate, Ag–Pd interdigital electrodes, and sensing materials. As prepared samples were ground into a fine powder and further mixed with distilled water and ethanol to form a homogeneous paste. This was subsequently screen printed onto the planar ceramic substrate to generate a uniform sensing film, and dried in air at 50 °C for 8 h. Finally, the fabricated gas sensor was aged at an aging test chamber for 12 days to improve the stability and repeatability before testing.

### 2.3. Gas Response Measurement

Gas sensing properties of the fabricated sensors to SOF_2_, SO_2_F_2_, and SO_2_ were measured and automatically recorded by a CGS-1TP (Chemical Gas Sensor-1 Temperature Pressure) intelligent gas sensing analysis system, purchased from Beijing Elite Tech Co., Ltd. (Beijing, China) [30]. Figure 1 shows a schematic diagram of the CGS-1TP system. The planar sensor was laid on the test chamber and its electrodes fixed by adjusting the two probes on each side to collect electrical signals. It is convenient to gain sensor resistance, gas response, working temperature, environmental temperature, and relative humidity with this system.

In this study, the gas response was defined as S = Ra/Rg, where Ra and Rg were the electrical resistance of the fabricated sensor in air and in the test gas, respectively. Response and recovery time was defined as the time required by the sensor to achieve 90% of the total resistance after injecting and removing the detected gas. All sensing measurements were tested under laboratory condition with room temperature (25 °C) and constant humidity (50% relative humidity), and repeated several times to ensure the reliability of the results.

## 3. Results and Discussion

### 3.1. Structural and Morphological Characterizations

**Figure 2:**
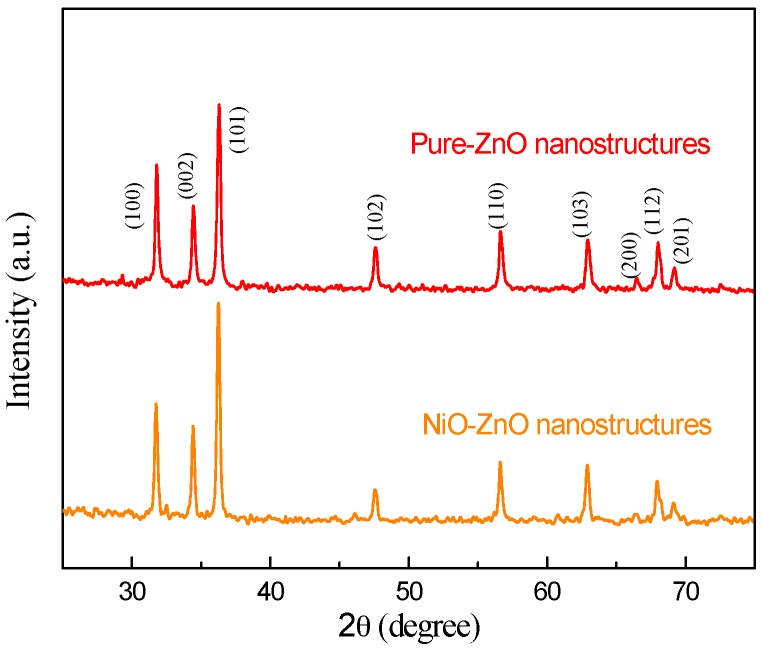
X-ray powder diffraction patterns of pure and 3 at-% NiO-decorated ZnO nanostructures.

The crystalline parameters of the as-prepared products were investigated by XRD and the results are presented in Figure 2. It is obvious that all of the peaks for both products can be well indexed and exactly match the standard pattern of ZnO (JCPDS. 36-1451). No obvious diffraction peaks of NiO were observed in the pattern, suggesting that the introducing of NiO did not change the original ZnO structure. It may be proposed that the amount of NiO is very small and it is highly dispersed in the ZnO samples. Moreover, the high peak intensities clearly indicate the high degree of crystallization of both of the samples, corresponding to a lower number of lattice defects [31]. No other doping diffraction peaks were observed from Figure 2, which suggest a high purity of our as-prepared products.

**Figure 3:**
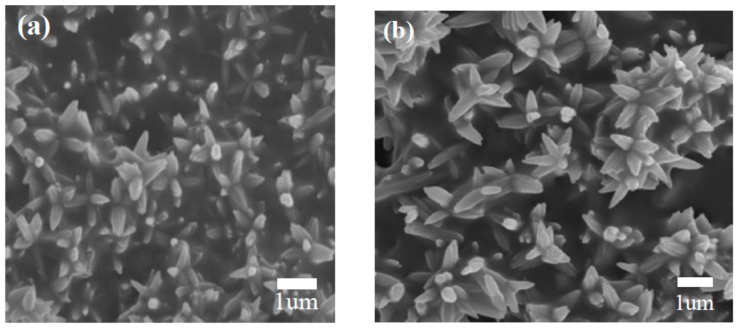

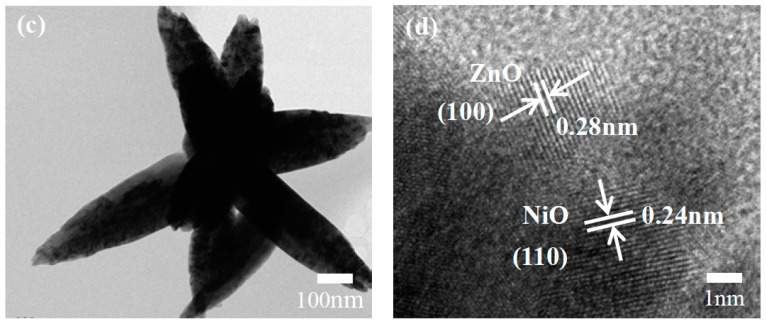
FESEM images of (**a**) pure and (**b**) NiO-decorated ZnO nanostructures (**c**) TEM and (**d**) HRTEM image of the NiO-decorated ZnO microflowers.

The overall surface morphologies of the pure and NiO-decorated ZnO nanostructures were firstly studied by FESEM. From Figure 3a,b it is clear that both products consist of a large amount of beautiful microflowers with smooth and approximately uniform size, and no other morphologies are observed. Comparing Figure 3a,b, the micro-morphological structures of the pure and NiO-decorated ZnO nanostructures are almost similar, which demonstrate that the introduction of NiO only has a slight impact on the morphology of pure ZnO products. Further morphology characterization was performed by TEM and shown in Figure 3c. The single microflower was composed of nanorods that connected to each other through the center to form 3D flower-like structures. This is in agreement with recently reported synthesized flower-like nanostructure [32]. The HRTEM image is shown in Figure 3d and the interplanar spacing of 0.28 nm as illustrated is consistent well with the (100) planes of ZnO (JCPDS. 36-1451). And the interplanar distances of 0.24 nm agree well with the lattice spacing of the (111) planes of the cubic NiO [33], which indicate that NiO has already exists in the composites.

**Figure 4:**
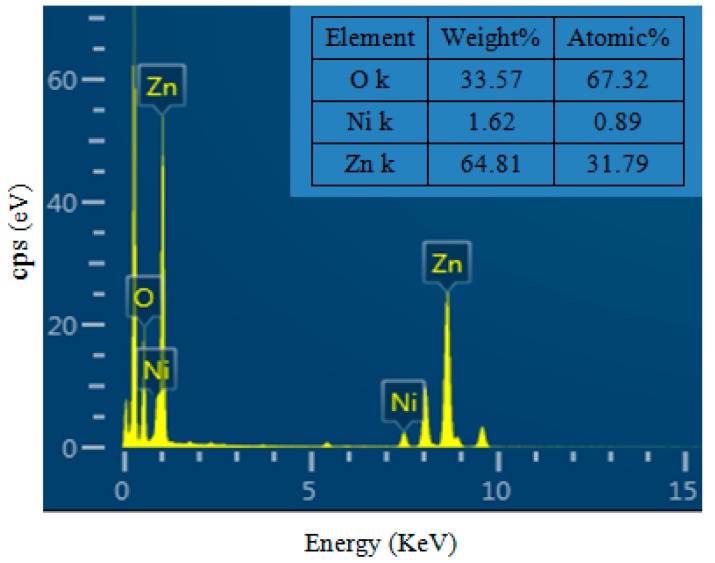
EDS spectra of 3 at-% NiO-decorated ZnO nanostructures.

In order to investigate the element components of the as synthesized samples, energy dispersive X-ray spectroscopy measurements were conducted. Figure 4 shows the EDS spectra of 3 at-% NiO-decorated ZnO products. It is clear that the elemental compositions of Ni, O and Zn, and the atomic percentage of Ni in the samples is calculated to be 2.72 at-%. Meanwhile no other EDS peaks have been found, which confirms our products are of high purity.

**Figure 5:**
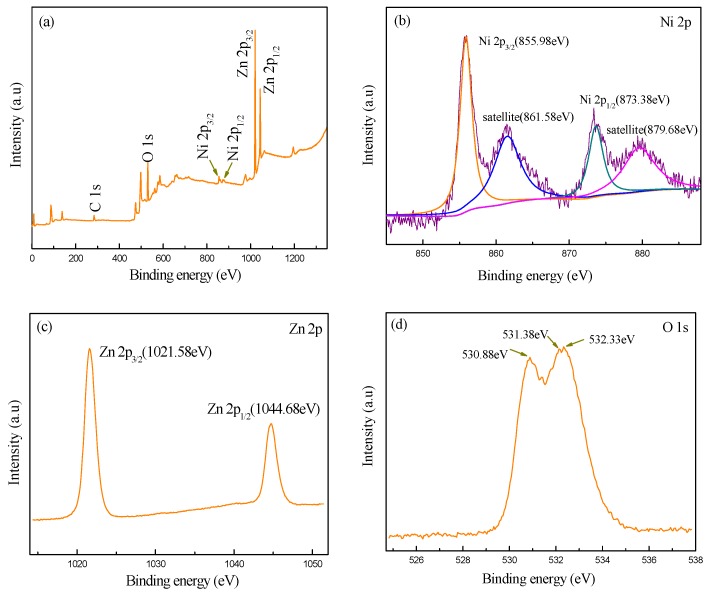
XPS survey spectra of NiO-decorated ZnO: (**a**) full spectrum; (**b**) Ni 2p; (**c**) Zn 2p; (**d**) O 1s.

The chemical compositions and chemical states of the samples were studied by X-ray photoelectron spectroscopy analysis, and the corresponding results are shown in Figure 5. As observed in Figure 5a, the XPS wide survey spectrum of the synthesized hierarchical samples confirms the existence of Zn, Ni, O, and C. It is proposed that C includes not only C compounds adsorbed by the samples from air, but also oily dirt from the apparatus. Figure 5b shows the high resolution scanning XPS spectra of Ni 2p. It is observed from the Ni 2p_3/2_ main peak and its satellite peak are located at 855.98 eV and 861.58 eV, respectively, while the Ni 2p_1/2_ main peak and its satellite peak appear at 873.38 eV and 879.68 eV, respectively. These results are in agreement with the previously reported values and further confirm the presence of NiO [34,35]. From Figure 5c, it is clear that the prominent peaks of Zn 2p_3/2_ and Zn 2p_1/2_ are at the binding energies of 1021.58 eV and 1044.68 eV. The observed spin-orbit splitting of Zn 2p between Zn 2p_3/2_ and Zn 2p_1/2_ is about 23 eV, which is consistent with the corresponding value of pure ZnO, indicating a normal state of Zn^2+^ in the synthesized sample [36]. In the spectra of O 1s as shown in Figure 5d, the peaks are located at 530.88 eV and 531.38 eV, and 532.33 eV respectively, which could be attributed to O^2−^ ions [37,38]. Therefore, it can be deduced that the Zn and Ni are present as ZnO and NiO in this sample, respectively.

### 3.2. Gas-Sensing Properties

**Figure 6:**
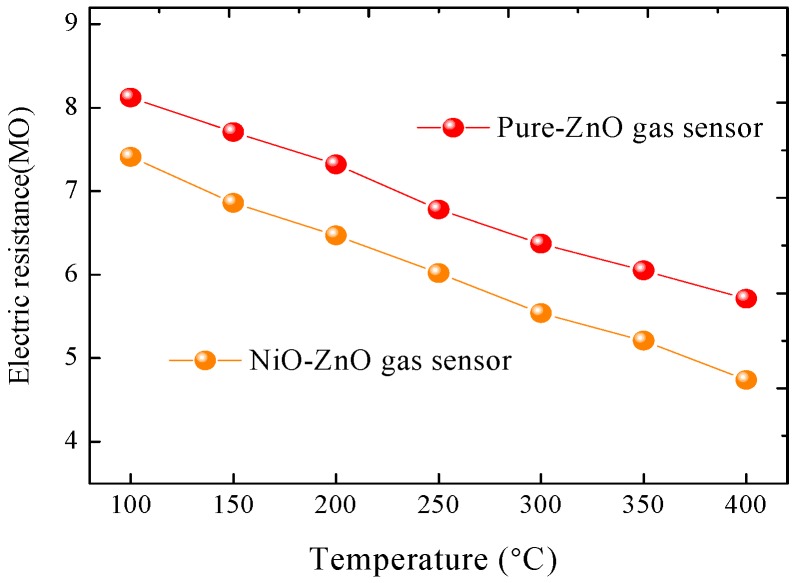
The electric resistance properties of the prepared sensors to different temperatures in air.

Figure 6 presents the electric resistance properties in pure air of the as prepared pure ZnO and NiO-decorated ZnO sensors at various operating temperatures from 100 °C to 400 °C. As seen in Figure 6, the resistance values of the two sensors decrease when the operating temperature increases, which is an intrinsic characteristic of a semiconductor gas sensor. Compared with pure ZnO sensor, the NiO-decorated ZnO sensor exhibits a higher resistance value at the same working temperature, which might be beneficial to the following gas sensing performances.

**Figure 7:**
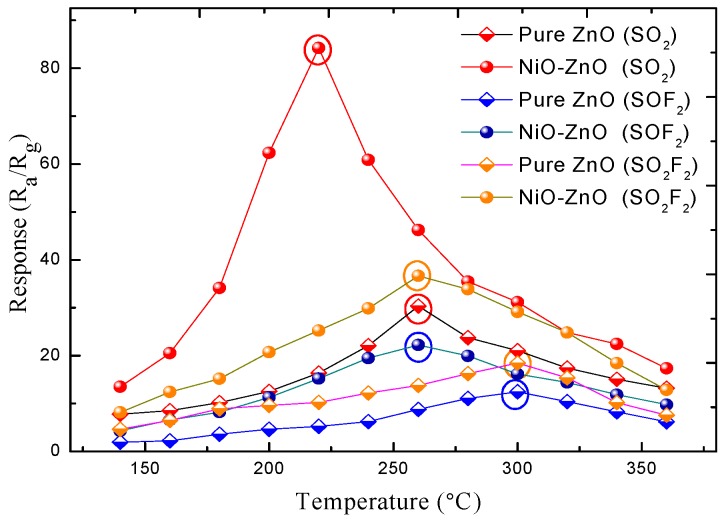
Gas response of the pure and NiO-decorated ZnO gas sensors to 100 ppm SO_2_, SOF_2_, and SO_2_F_2_ at different working temperature.

In general, the sensing performance of fabricated gas sensors is strongly influenced by the operating temperature. In order to demonstrate that the addition of NiO nanoparticles is an effective way to enhance the gas sensing properties of ZnO-based gas sensor, the effects of various different operating temperatures ranging from 140 °C to 360 °C on fabricated sensors to 100 ppm of SO_2_, SOF_2_, and SO_2_F_2_ were respectively investigated and illustrated in Figure 7. It is found that the response of the pure and NiO-decorated ZnO sensors to SO_2_ firstly improve with increasing working temperatures and gave the largest values of about 30.35 at 260 °C and 84.26 at 220 °C, respectively, then it decreased with further rising temperatures. Similar sensing behaviors and gas response curves are observed for SOF_2_ and SO_2_F_2_. The maximum gas response of the pure and NiO-decorated ZnO sensors against SOF_2_ appear at about 300 °C and 260 °C with responses 12.49 and 22.25, respectively. The maximum response values to SO_2_F_2_ are about 18.46 at 300 °C and 36.67 at 260 °C for the pure and NiO-decorated ZnO sensors. Comparison of these sensing results indicate that the introduction of NiO can not only reduce the optimum operating temperature, but also enhance the gas response to SF_6_ decomposed components, along with obvious changes in the temperature characteristic curve.

**Figure 8:**
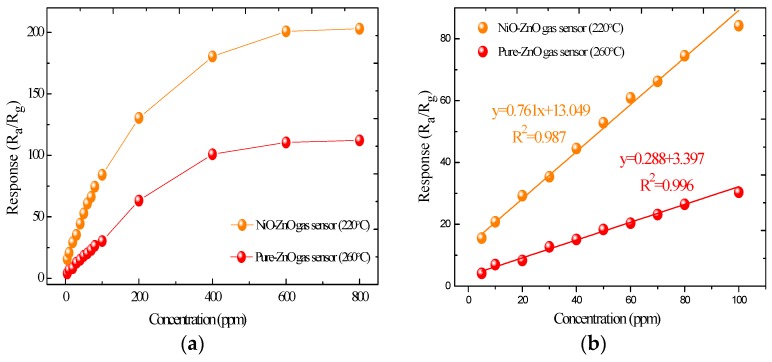
(**a**) Response of the pure and NiO-decorated ZnO gas sensors to various concentrations of SO_2_ at 220 °C; (**b**) Linear fitting curves of pure and NiO-decorated ZnO sensors to 5~100 ppm of SO_2_.

Figure 8 depicts the gas responses of the pure and NiO-decorated ZnO sensors to SO_2_ at different concentrations at their respective optimum operating temperature. From Figure 8a, the response continuously increases with gas concentrations ranging from 5 to 800 ppm. Moreover, it is clear that the NiO-decorated ZnO sensor demonstrates enhanced sensing properties for SO_2_ detection compared with the pure ZnO sensor. Figure 8b exhibits a linear calibration curve of the sensors with SO_2_ concentration in the range of 5~100 ppm. In this area, the linear fitting functions of pure and NiO-decorated ZnO gas sensors are y = 0.288x + 3.397 and y = 0.761x + 13.049, respectively. Their linear correlation coefficients R^2^ are as high as 0.996 and 0.987, which indicates that the response of SO_2_ concentration variation at low concentration is close to linear.

**Figure 9:**
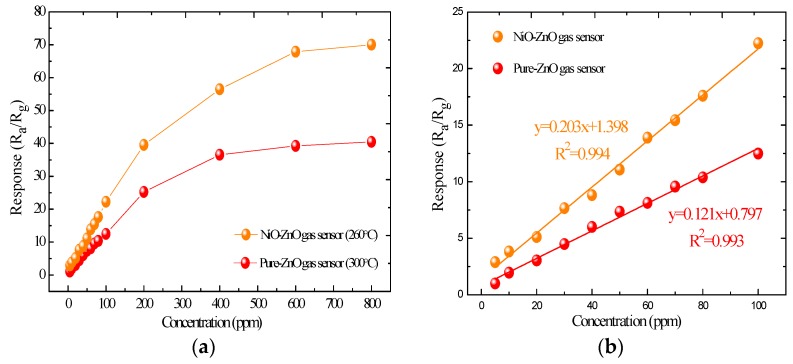
(**a**) Pure and NiO-decorated ZnO gas sensors’ response to different concentrations of SOF_2_ at the 260 °C operating temperature; (**b**) Linear relationship between the sensors’ response value and the SOF_2_ concentration.

The gas responses of the pure and NiO-decorated ZnO sensors to SOF_2_ with different concentrations at their respective optimum working temperatures were measured and are shown in Figure 9. The responses of the fabricated sensors increase rapidly in linearity with increasing SOF_2_ concentration below 200 ppm, but increase much more slowly above 200 ppm. Moreover, we can also find that the response of the NiO-decorated ZnO sensor is nearly two times higher, on average, than that of the pure ZnO sensor. As Figure 9b exhibits, the linear relationship of the pure and NiO-decorated ZnO sensors between the sensing response and gas concentration in the range of 5~100 ppm are fitted by the equation y = 0.121x + 0.797 and y = 0.203x + 1.398, with high liner correlation coefficients R^2^ of 0.993 and 0.994, respectively.

**Figure 10:**
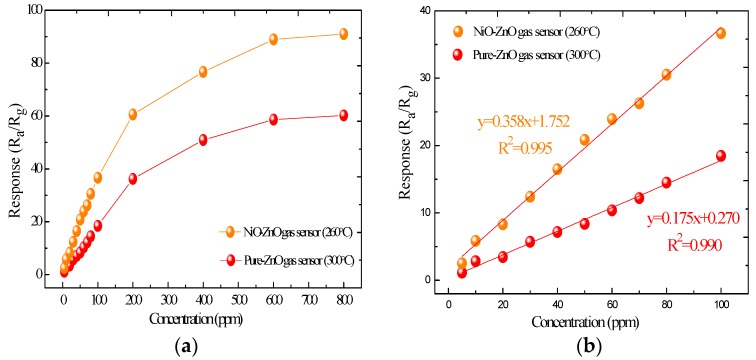
(**a**) Response of the pure and NiO-decorated ZnO gas sensors to SO_2_F_2_ with different concentration at 260 °C; (**b**) Linear fitting curves of the prepared the sensors to 5~100 ppm of SO_2_F_2_.

Figure 10 shows the responses of the pure and NiO-decorated ZnO sensors as a function of SO_2_F_2_ with different concentrations at their respective optimum operating temperatures. As Figure 10a shows that the gas responses of the sensors increase rapidly with increasing SO_2_F_2_ concentration, but increase much more slowly with the further increase of the SO_2_F_2_ concentration and nearly reach their saturation at about 600 ppm. Furthermore, the response of the NiO-decorated ZnO sensor is higher than that of the pure one. From Figure 10b the linear fitting functions of pure and NiO-ZnO gas sensors to 5~100 ppm of SO_2_F_2_ are y = 0.175x + 0.270 and y = 0.358x + 1.752, respectively. And their linear correlation coefficients R^2^ were calculated to be 0.990 and 0.995, respectively.

According to the sensing results mentioned above, it can be concluded that the gas responses of the NiO-decorated ZnO sensor to SO_2_, SO_2_F_2_ and SOF_2_ is higher than that of the pure one. Moreover, the responses of the prepared sensors to these three test gases at low concentration have a good linear relationship. These good linear relationships indicate that the fabricated gas sensors are more likely to be used in practice [39,40].

**Figure 11:**
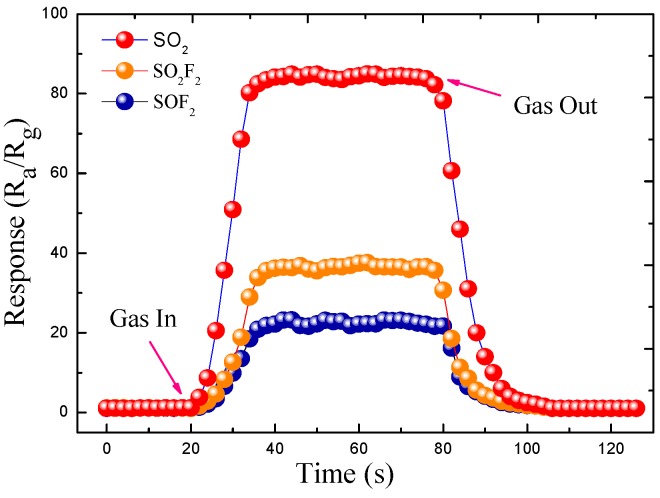
Response-recovery curves of the NiO-decorated ZnO gas sensor to 100 ppm SO_2_, SO_2_F_2_ and SOF_2_.

Figure 11 presents the response and recovery curves of the NiO-decorated ZnO sensor to 100 ppm SO_2_, SO_2_F_2_ and SOF_2_ working at their respective optimum operating temperature. As seen in Figure 10, the common feature of these three test gases is that the response of the sensor increases rapidly when gas is injected into the gas chamber for sensing and dramatically decreases to its initial value when the gas is removed. According to the definition above, the response times to 100 ppm SO_2_, SO_2_F_2_ and SOF_2_ were calculated to be about 12, 16 and 18 s, while the recovery times were calculated to be about 16, 20 and 22 s, respectively.

**Figure 12:**
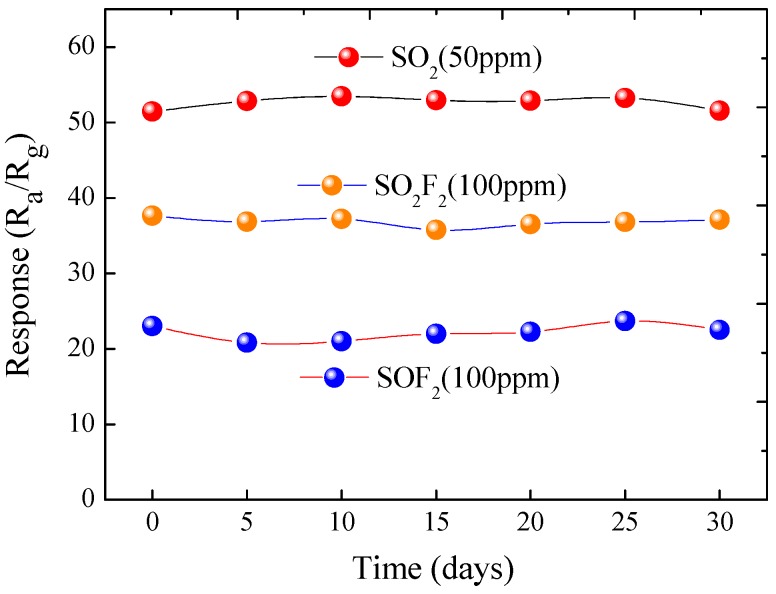
The stability and repeatability of the NiO-decorated ZnO sensor against SO_2_, SO_2_F_2_ and SOF_2_.

Finally, the long-time stability and repeatability of the NiO-decorated ZnO sensor to 100 ppm SO_2_, SO_2_F_2_ and SOF_2_ at their respective optimum working temperatures were measured and shown in Figure 12. As the figure shows, all of the gas responses change slightly and remain at a nearly constant value during the long experimental cycles, which indicates the excellent long-term stability and repeatability of the fabricated sensors for detecting these three kinds of SF_6_ decomposition byproducts.

### 3.3. Sensing Mechanisms

The sensing mechanism for ZnO, a typical n-type semiconductor gas sensing material, has been interpreted in some former papers [41,42]. It is believed that the sensing properties of ZnO chemical gas sensors are mainly determined by the change of electric resistance, which is fundamentally attributed to the chemical adsorption and desorption process of target gas molecules on the surface of gas sensing materials [43]. In an air atmosphere, oxygen could be absorbed on the ZnO surface acting as a trap capturing electrons from its conduction band to form a depletion region on the surface. This depletion region would lead to an increase of the sensor resistance. When exposed to a target gas, the test gas molecules react with the adsorbed oxygen and the trapped electrons are released back into the conduction band, and thus as decreased resistance is measured [44]. According to the definition of gas response (Ra/Rg) the response of the sensor increases.

It was observed in the present study that the sensing properties of ZnO were greatly enhanced due to the introduction of NiO. Two main reasons satisfactorily explain this interesting performance. It has been acknowledged that the gas sensing properties of semiconductor nanomaterials with different morphologies are different [45]. In this study, the synthesized flower-like nanomaterials offer a larger surface accessibility, which will be favorable to adsorption and desorption of target gas molecules, and this unique morphology is helpful to improve the gas sensing response. Moreover, the formation of p–n junctions between NiO and ZnO in the grain boundaries results in an enhancement of gas sensing. A hetero p–n junction system has been proposed to explain the enhanced gas response of Co_3_O_4_-WO_3_ [46], NiO-SnO_2_ [47], LaFeO_3_-SnO_2_ [48] heterocontacts.

**Figure 13:**
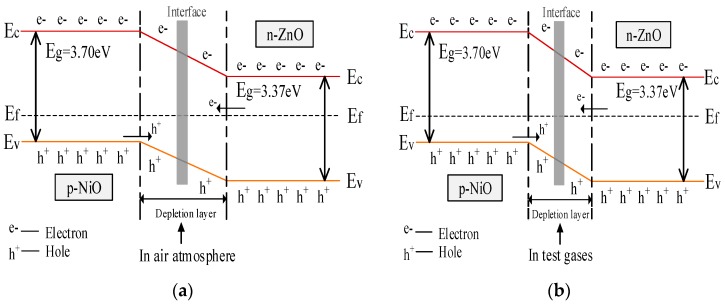
Energy band schematics for p-type NiO/n-type ZnO heterojunction (**a**) in air atmosphere; (**b**) in test gases. Ec: lower level of conduction band; EF: Fermi level; Ev: upper level of valence band.

As we all know, ZnO shows n-type conductivity by electrons and NiO shows p-type conductivity by holes. By introducing NiO into ZnO, the electrons in ZnO and holes in NiO transport in opposite directions until the system reaches equalization of the Fermi levels, leading to the formation of a p–n junction, as shown in Figure 13a [49]. This is the thermal equilibrium of the formation of a p–n junction [50]. When the composite was exposed to air, oxygen molecules can absorb on the surface of the material and form chemically adsorbed oxygen. With different operating temperatures chemisorbed oxygen exists in various forms [51], as shown in the reaction Equations (1)–(4).

O_2_ (gas) → O_2_ (ads) (1)

O_2_ (gas) + 2e^−^ → 2O^−^ (ads) (2)

½ O_2_ (ads) + 2e^−^ → O^2−^ (ads) (3)

O_2_ (ads) + e^−^ → O_2_^−^ (ads) (4)

Moreover, the resistance of the NiO-decorated ZnO in air (Ra) will be even higher than without the heterojunctions (pure ZnO) due to a new depletion layer between NiO and ZnO [33,52]. Nevertheless, when the composite was exposed to test gases (SO_2_, SOF_2_ and SO_2_F_2_), the gas molecules react with the absorbed oxygen species of gas sensing material and release the electrons back to the material. The three SF_6_ decomposed components all play the role of an electron donating gas [53]. The reaction equation is as follows:

R + O^−^ (ads) «═» RO (ads) + e^−^ (5)

where R is one of SF_6_ decomposition component gases, and O^−^(ads) denotes the adsorbed oxygen on the material’s surface. Owing to the electron-hole recombination, the hole concentration of p-type NiO decreases, and as a result the concentration gradient of the p–n junction is reduced. Thus, the effect of carriers transport becomes weak and the depletion layer becomes thin [50,54], as shown in Figure 13b. The resistance of the sensor in the test gas is further decreased [55]. In short, compared with a pure ZnO sensor, the formation of p–n junctions greatly increase the resistance of the ZnO sensor in air and further decreases the resistance in the test gas. Thus, based on the definition of gas response (Ra/Rg), the responses of the gases are greatly enhanced due to the variation of resistance [49].

## 4. Conclusions

In summary, pure and 3 at-% NiO-decorated ZnO sensing materials were successfully synthesized via a one-step hydrothermal method and characterized by XRD, FESEM, TEM, HRTEM, EDS and XPS, respectively. Their gas sensing properties toward the SF_6_ decomposition byproducts SO_2_, SO_2_F_2_, and SOF_2_ were systemically researched with a CGS-1TP gas sensing analysis system. Comparative results demonstrate that the as prepared NiO-decorated ZnO sensing material exhibits enhanced gas sensing properties, including lower optimal working temperature, higher gas response and shorter response-recovery time. Such good performance is attributable to the formation of p–n junctions between NiO and ZnO. This work provides an effective way for designing high-performance gas sensors to detect SF_6_ decomposition byproducts like SO_2_, SO_2_F_2_, and SOF_2_.
